# A New Proton Therapy Solution Provides Superior Cardiac Sparing Compared With Photon Therapy in Whole Lung Irradiation for Pediatric Tumor Patients

**DOI:** 10.3389/fonc.2020.611514

**Published:** 2021-02-02

**Authors:** Xue Sha, Jinghao Duan, Xiutong Lin, Jian Zhu, Ruohui Zhang, Tao Sun, Hui Wang, Xiangjuan Meng, Yong Yin

**Affiliations:** ^1^ Department of Radiation Oncology, Shandong Cancer Hospital and Institute, Shandong First Medical University and Shandong Academy of Medical Sciences, Jinan, China; ^2^ Shandong Provincial Key Laboratory of Digital Medicine and Computer-Assisted Surgery, Qingdao, China; ^3^ Department of Radiation Oncology, The Fourth Hospital of Hebei Medical University, Shijiazhuang, China; ^4^ Department of Radiation Oncology, Qingdao Central Hospital, Qingdao, China

**Keywords:** proton radiotherapy, cardiac sparing, whole lung irradiation, pediatric tumor, photon radiotherapy

## Abstract

**Objective:**

Whole lung irradiation (WLI) plays a crucial role in local control in pediatric patients with lung metastases and improves patient survival. The intention of this research was to explore the advantage of cardiac sparing between photons and protons during WLI. We also propose a new solution for cardiac sparing with proton techniques.

**Methods:**

Eleven patients with pediatric tumors and pulmonary metastasis treated with 12 Gy WLI (all received volumetric-modulated arc therapy (VMAT)) in our institute between 2010 and 2019 were retrospectively selected. Each patient was replanned with intensity-modulated radiation therapy (IMRT), helical tomotherapy (HT), and two intensity-modulated proton radiotherapy (IMPT) plans (IMPT-1 and IMPT-2). IMPT-1 considered the whole lung as the planning target volume (PTV), utilizing the anteroposterior technique (0/180°). IMPT-2 was a new proton solution that we proposed in this research. This approach considered the unilateral lung as the PTV, and 3 ipsilateral fields were designed for each lung. Then, IMPT-2 was generated by summing two unilateral lung plans. The primary objective was to obtain adequate coverage (95% of the prescription dose to the PTV) while maximally sparing the dose to the heart. The PTV coverage, conformity index (CI), homogeneity index (HI), and dose–volume statistics of the heart and substructures were assessed by means of the averages of each comparison parameter.

**Results:**

All treatment techniques achieved the target volume coverage required by clinical practice. HT yielded the best coverage and homogeneity for the target structure compared with other techniques. The CI from IMRT was excellent. For photon radiation therapy, the HT plan afforded superior dose sparing for the V_5_, V_6_, V_7_, V_8_, and D_mean_ of the heart and D_mean_ of the right ventricle (RV). IMRT displayed the most notable dose reductions in the V_9_, V_10_, V_11_, and V_12_ of the heart and D_mean_ of the right atrium (RA). The VMAT plan was the least effective on the heart and substructures. However, compared with photon radiation therapy, IMPT-1 did not show an advantage for heart protection. Interestingly, IMPT-2 provided significant superiority in cardiac sparing, including maximum dose sparing for the V_5_, V_6_, V_7_, V_8_, V_9_ and D_mean_ of the heart and D_mean_ of the RA, RV, left atrium (LA) and left ventricle (LV) compared to all other techniques.

**Conclusions:**

Considering the complex anatomical relation between target volumes and organs at risk (OARs), IMPT can provide a dose advantage for organs located outside of the target area rather than within or surrounding the area. It is hoped that advances in proton therapy (PT) plan design will lead to further improvements in radiotherapy approaches and provide the best treatment choice for individual patients.

## Introduction

Malignant tumors are the second leading cause of death in children, with over 300,000 new cases diagnosed annually (1, 2). The lung is the most common site of metastasis, with approximately 20 to 25% of patients with Wilms tumor or Ewing sarcoma showing metastatic lesions on chest radiography at diagnosis (3). Whole lung irradiation (WLI) plays a crucial role in local control in patients with lung metastases, those who had incomplete resection, and those with an unfavorable histology, advanced stage, and high-risk chromosomal aberrations (4, 5). Published studies have indicated that WLI is an essential component in the current multimodality treatment and can achieve a survival rate of 90% (6–8).

With the increased proportion of survivors, the risk of late toxicities resulting from a combination of radiotherapy and toxic cardiac chemotherapy is becoming increasingly concerning. Advances in imaging science and radiotherapy technology have allowed precise tumor determination and delineation and high conformity to the target volume. However, due to the non-targeted radiation dose, the surrounding normal tissue is still at risk. Cardiac toxicity is a common delayed effect observed in pediatric patients after chemotherapy and WLI. Studies have revealed that in child survivors, WLI has led to a high prevalence of a variety of cardiac complications, including vascular heart disease, myocardial infarction, congestive heart failure (CHF) and pericardial disease (9). WLI has traditionally been combined with standard anterior/posterior field photon irradiation, resulting in poor heart-sparing potential. Therefore, to achieve a lower dose to the heart, new techniques, such as multiple field intensity-modulated radiation therapy (IMRT), volumetric-modulated arc therapy (VMAT), and helical tomotherapy (HT), are desirable. Although these techniques contribute to the reliable treatment delivery of the radiation dose to the diseased tissues, the crucial problem of overdose to the heart during treatment remains unsolved.

Proton therapy (PT), as a frontier radiotherapy technique, offers distinct physical properties that can contribute to an improvement in dose distribution with a subsequent reduction in the integral dose to the patient, supporting the potential value of proton beams in tumors close to the target volume. A series of studies have demonstrated that children with malignant tumors have good tolerance to proton beams, and this plan ensures good tumor control probability, prolonged survival, intelligence quotient protection and reduced risk of a secondary tumor (10). Consequently, we hope for an advantage of PT over photon therapy that will lead to improved indications for WLI. However, with the current proton treatment planning system (TPS), PT cannot provide an advantage for cardiac sparing in WLI. For this reason, we propose a new solution for cardiac sparing in proton techniques, and we expect this solution to reduce the exposure dose to the heart and diminish the complications associated with radiation-induced cardiac injury in pediatric patients receiving WLI. Our research may lead to improvements in the PT TPS and provides useful guidelines for selecting reasonable treatment techniques in WLI.

## Materials and Methods

### Patient Eligibility

Eleven patients (median age, 3 years; range 2–17 years) with different histologies (five with Wilms tumor, three with rhabdomyosarcoma, two with Ewing sarcoma, and one with germ cell tumor) who received WLI in our institute between January 1, 2010 and December 31, 2019, were retrospectively selected. The retrospective analysis of the medical records was approved by the Institutional Review Board of Shandong Cancer Hospital. The characteristics of the patients are displayed in **Table 1**.

**Table 1:** Patient characteristics.

Patient	Diagnosis	Primary site	Age at diagnosis/Sex	Stage	Dose (Gy)/fractions
1	Ewing sarcoma	Astragalus	11/Female	IV	12/10
2	Wilms tumor	Left kidney	2/Female	IV	12/10
3	Wilms tumor	Right kidney	3/Female	IV	12/10
4	Wilms tumor	Left kidney	7/Male	IV	12/10
5	Rhabdomyosarcoma	Arm	17/Male	IV	12/10
6	Rhabdomyosarcoma	Left kidney	3/Female	IV	12/10
7	Rhabdomyosarcoma	Abdomen	3/Male	IV	12/10
8	Wilms tumor	Right kidney	3/Male	IV	12/10
9	Wilms tumor	Right kidney	13/Male	IV	12/10
10	Germ cell tumor	Sacrococcyx	3/Female	IV	12/10
11	Ewing sarcoma	Astragalus	3/Female	IV	12/10

### Target Volume and Organs at Risk Definitions

Patients were placed in a customized site-specific immobilization device for the treatment position, and computed tomography (CT) simulation provided images at 3 mm for both lungs. For patients who could not cooperate with positioning, chloral hydrate was injected to produce a sedative hypnotic effect, ensuring a precise posture. The target volume, clinical target volume (CTV), was defined as total lung extension from the apex to the diaphragm using the acquisition window/level setting. The planning target volume (PTV) was delineated by expanding the 0.3 to 0.5 cm margin of the CTV. For inverse planning techniques, a heart-PTV structure that consisted of the volume overlap of the heart and the PTV was created to enhance the optimization process. OARs considered in the present study included the esophagus, liver, spinal cord, vertebral column, humerus, heart, right atrium (RA), right ventricle (RV), left atrium (LA) and left ventricle (LV) (contoured using the Radiation Therapy Oncology Group contouring atlas).

### Treatment Planning

**Figure 1:**
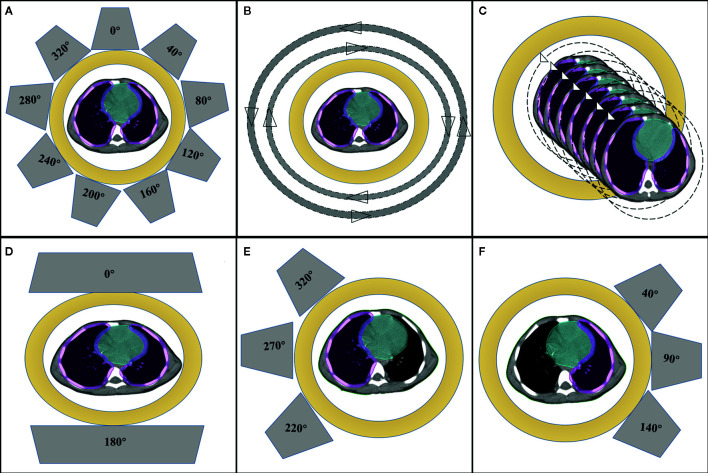
Beam arrangements for **(A)** IMRT, **(B)** VMAT, **(C)** HT, **(D)** IMPT-1, **(E)** sum and **(F)** IMPT-2.

The prescribed dose was 12 Gy in all patients, and the daily fraction dose was 1.2 Gy. The primary objective was to obtain adequate coverage (95% of the prescription dose to the PTV) while maximally sparing the dose to the OARs, especially the heart and substructures. For each patient, five plans were created: IMRT, VMAT, HT, IMPT-1, and IMPT-2. All plans were generated by senior radiation physicists with more than ten years of experience designing radiotherapy plans. The beam arrangements are shown in **Figure 1**.

IMRT plans were performed with a Varian Trilogy linear accelerator using beam energies of 6 MV photons and beam angles of 0, 40, 80, 120, 160, 200, 240, 280 and 320° for cardiac sparing. The sliding window technique, by having the leaf pairs move across the field at a variable rate, was used to deliver the nine-field modulated plan on the Eclipse 13.6 TPS (Varian Medical Systems, Palo Alto, CA). The field sizes and weights of a series of beam segments were determined by iterative, automated optimization techniques. For the VMAT technique, two full arcs were delivered to 10 patients: a clockwise arc traveling from 181 to 179° and a counterclockwise arc traveling from 179 to 181°. The oldest patient with the largest lung volume required three full arcs. A collimator angle of 10° for the clockwise arc and 350° for the counterclockwise arc were used. HT plans were created with the Tomotherapy version 5.1.3 TPS using a HiArt unit (Accuray^®^ Planning Station, Madison, WI, USA). In general, the parameters specified as part of the optimization process were the field width, pitch, and modulation factor. In the current research, a pitch of 0.287, a collimator width of 2.5 cm, and a modulation factor of 2.4 were selected.

Two IMPT plans were generated in the Varian Eclipse ProBeam proton system and used for multiple field optimization and selective robust optimization. IMPT-1 considered the whole lung as the PTV, utilizing the anteroposterior technique (0/180°). IMPT-2 included the sum of two IMPT plans that considered the unilateral lung as the PTV. For the left lung, the IMPT plan was designed with three fields, with gantry rotations of 40, 90, and 140°. For the right lung, the IMPT plan was designed with three felids, with gantry rotations of 220, 270, and 320°. For individual patients, the gantry rotation was adjusted to minimize the exposure to the heart as much as possible. The proton dose was determined using a relative biologic effectiveness (RBE) of 1.1 and is specified in cobalt gray equivalent (CGE) units (11). The non-linear universal proton optimizer (NUPO) algorithm was used to generate the plan, and the dose was calculated with the proton convolution superposition algorithm with a grid size of 0.25 cm. A positioning error of 3 mm and a range uncertainty of ±3% were taken into account during planning optimization.

### Treatment Plan Analysis

Dose–volume data for the PTV and OARs obtained from dose-volume histograms (DVHs) were determined for each technique from the 11 scans. The following dosimetry parameters for the PTV were evaluated: target coverage, dose received by 2% of the target volume (D_2%_), dose received by 98% of the target volume (D_98%_), maximum dose (D_max_), medial dose (D_mean_), minimum dose (D_min_), conformity index (CI), and homogeneity index (HI). The CI was calculated according to the following expression (12):

$$\text{CI}=\frac{TV_{RI}}{TV}\times \frac{TV_{RI}}{V_{RI}}$$

where TV_RI_ is the target volume covered by the prescription isodose, TV is the target volume, and V_RI_ is the volume of the prescription isodose. The CI ranged from 0 to 1, where 1 indicated perfect overlap (identical structures). A value near 0 indicated the total absence of conformation, *i.e.*, the target volume was not irradiated.

$$\text{HI}=\frac{D_{2\%}-D_{98\%}}{D_{prescription}}$$

where D_prescription_ is the prescription dose of the target volume. The HI ranged from 0 to 1, where 0 was the ideal value. A higher HI indicates poorer homogeneity.

The following dosimetric parameters were evaluated for the heart: V_5_, V_6_, V_7_, V_8_, V_9_, V_10_, V_11_, and V_12_ (V_X_ represents the volume percentage receiving more than x Gy) and D_mean_. Additional parameters analyzed included D_mean_ for the RA, RV, LA and LV. Additionally, to evaluate dose delivery efficiency, monitor units (MUs), control points (or segments) per fraction and beam on time were compared.

### Statistical Analysis

The Wilcoxon matched-pairs signed-rank test was used to compare the dose differences between different radiotherapy techniques. Data analysis was performed with MATLAB software version R2018a (MathWorks, Chicago, IL, USA). *P*-values<0.05 were considered statistically significant.

## Results

### Comparison of Target Volume Dosimetry

**Figure 2:**
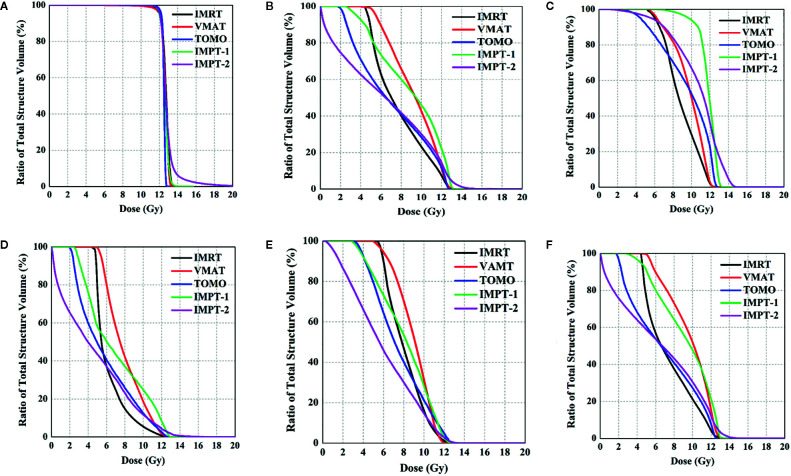
Comparison of DVHs for the PTV and OARs. **(A)** PTV, **(B)** whole heart, **(C)** right atrium, **(D)** right ventricle, **(E)** left atrium, and **(F)** left ventricle.

All treatment techniques achieved the target volume coverage required by clinical practice. HT plans yielded the best coverage for the target structure, with 98% (range 97–99%) of the PTV receiving 95% of the prescribed dose. Nevertheless, the coverage of the target volumes was equivalent between the IMRT, VMAT, IMPT-1 and IMPT-2 plans, and no significant difference was found in the present study. The maximum target dose was achieved with the IMPT-2 plan, and the minimum target dose was achieved with the VMAT plan. In general, the mean target doses were compromised, and all techniques resulted in similar D_mean_ values. Moreover, the CI was excellent with the IMRT plan, demonstrating the best consistency between the target volume and the shape of the radiation fields in the treatment delivery. Additionally, HT plans were associated with a favorable HI and reflected uniform dose distributions. Comparative dosimetry of the target volumes for five plans is reported in **Table 2**, and the cumulative DVHs of the PTV are shown in **Figure 2A**.

**Table 2:** Summary of the target volume dosimetry.

	IMRT	VMAT	HT	IMPT-1	IMPT-2	P < 0.05
D_2_(Gy)	13.2 ± 0.1	13.1 ± 0.07	12.7 ± 0.1	13.0 ± 0.4	14.74 ± 1.24	a,b,d,f,g,h,i,j
D_98(_Gy)	11.8 ± 0.1	11.8 ± 0.2	12.0 ± 0.1	11.9 ± 0.5	11.84 ± 0.09	b,f, g,h,j
D_max_(Gy)	14.1 ± 0.3	13.6 ± 0.2	13.0 ± 0.1	14.6 ± 0.7	21.81 ± 3.93	a,b,c,d,e,f,g,h,i,j
D_mean_(Gy)	12.6 ± 0.03	12.6 ± 0.007	12.5 ± 0.1	12.6 ± 0.001	12.75 ± 0.14	b,d,f,g,h,i,j
D_min_(Gy)	8. 4 ± 1.2	5.6 ± 2.0	10.2 ± 0.4	6.6 ± 2.4	6.31 ± 1.72	a,b,c,d,e,f, g,i
Target coverage(%)	96.2 ± 0.4	96.2 ± 1.3	98.2 ± 0.5	96.0 ± 0.8	96.36 ± 1.03	b,f,i
CI	0.86 ± 0.02	0.84 ± 0.03	0.84 ± 0.03	0.84 ± 0.03	0.80 ± 0.05	b,g,j
HI	0.1 2 ± 0.01	0.11 ± 0.02	0.05 ± 0.01	0.10 ± 0.07	0.24 ± 0.11	b,d,f, g,h,i,j

IMRT, intensity-modulated radiation therapy; VMAT, volumetric-modulated arc therapy; HT, helical tomotherapy; IMPT, intensity-modulated proton therapy; Dx, dose received by x% of the volume; CI, conformity index; HI, homogeneity index; a, IMRT vs VMAT; b, IMRT vs HT; c, IMRT vs PT; d, VMAT vs HT; e, VMAT vs PT; f, HT vs PT; g, IMRT vs IMPT-2; h, VMAT vs IMPT-2; I, HT vs IMPT-2; j, IMPT-1 vs IMPT-2.

### Cardiac Sparing

IMPT-2 resulted in marked cardiac sparing, yielding the lowest D_mean_ of the heart and substructures, the most focused dosimetric parameters of the heart. The D_mean_ values of the whole heart, RA, RV, LA and LV in the IMPT-2 plan were 5.5 ± 0.9, 8.3 ± 1.1, 2.8 ± 1.3, 4.2 ± 1.0, and 8.3 ± 1.1, respectively. Moreover, statistical analysis indicated significant differences between this plan and the other plans (*P*-values less than 0.05). ATIThis treatment planning study demonstrates that PT delivers higher tumor doses than photon therapy while sparing normal tissues.

**Figure 3:**
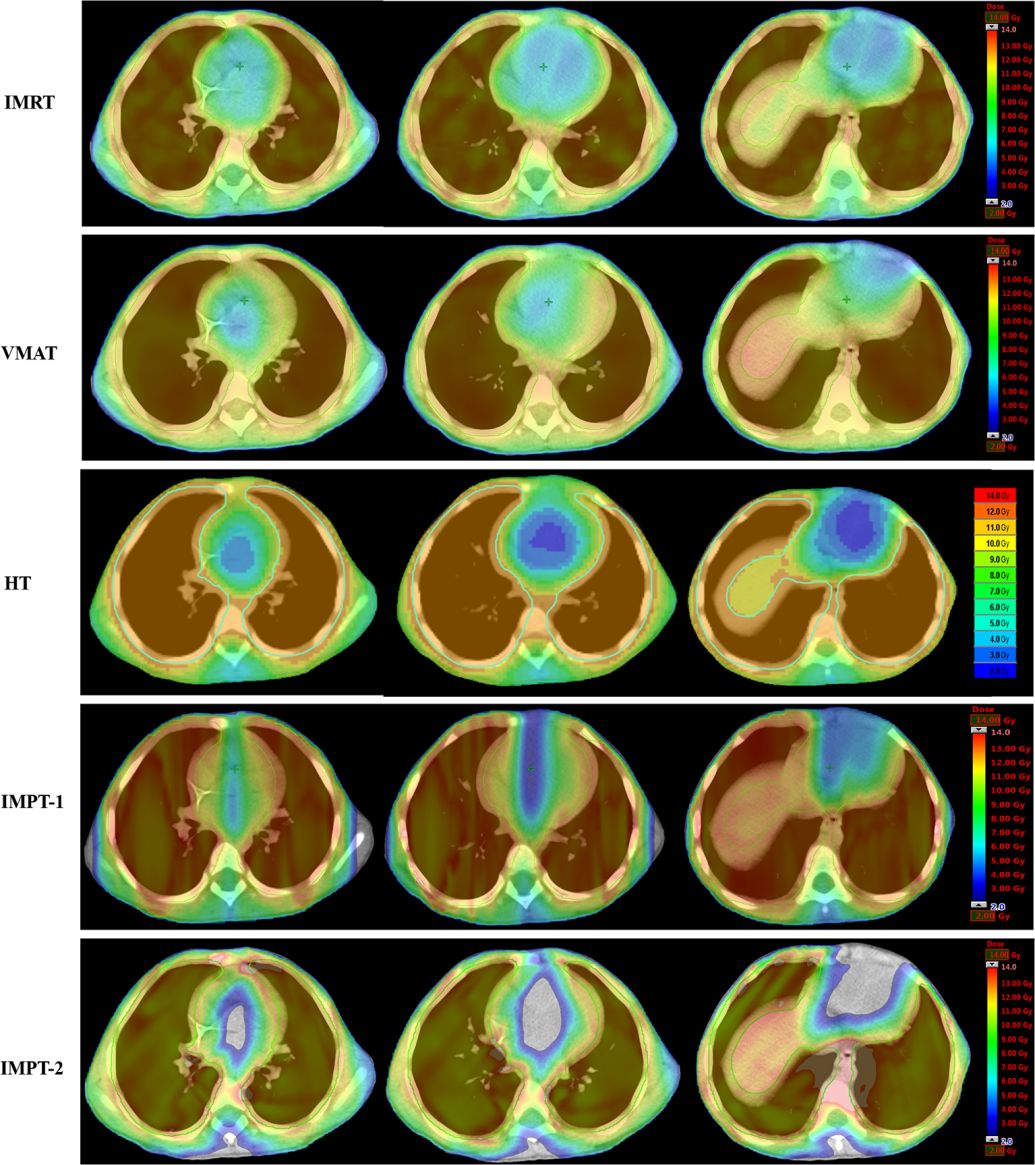
Color wash of the absorbed-dose distribution (transverse plane) to the heart in four-year-old male patients.

For the photon plan, HT afforded superior dose sparing for the V_5_, V_6_, V_7_, V_8,_ and D_mean_ of the heart, while the greatest reductions in the V_9_, V_10_, V_11_, and V_12_ of the heart were observed with the IMRT plan. Concerning cardiac structures, IMRT resulted in the most notable dose reduction to the RA, and HT displayed the most notable dose reduction to the RV. However, VMAT showed the poorest reduction in various dosimetric parameters of the heart. **Table 3** summarizes the various absorbed-dose parameters for cardiac structures, and cumulative DVHs are shown in **Figures 2B–E**. **Figure 3** shows schematic diagrams of the absorbed-dose distribution for the heart.

**Table 3:** Dose–volume histogram (DVH) statistics for cardiac structures.

Heart	IMRT	VMAT	HT	IMPT-1	IMPT-2	P<*0.05*
V_5_ (%)	79.6 ± 27.5	82.3 ± 27.7	60.1 ± 19.2	66.1 ± 23.0	44.5 ± 16.32	b,c,d,e,g,h,i,j
V_6_ (%)	66.6 ± 12.2	80.4 ± 13.5	55.8 ± 4.0	62.4 ± 7.8	43.37 ± 8.46	b,d,e,g,h,i,j
V_7_ (%)	53.7 ± 13.0	69.4 ± 14.6	47.5 ± 4.1	52.8 ± 7.0	38.69 ± 7.85	a,d,e,f,g,h,i,j
V_8_ (%)	43.1 ± 11.7	58.5 ± 15.5	40.2 ± 4.0	49.4 ± 6.4	33.94 ± 6.99	a,c,d,f,h,j
V_9_ (%)	33.6 ± 10.1	48.8 ± 17.2	33.7 ± 4.0	43.0 ± 6.0	29.21 ± 6.26	a,c,d,f,h,j
V_10_ (%)	24.7 ± 7.4	38.9 ± 18.9	27.2 ± 3.8	36.1 ± 5.9	24.11 ± 5.45	a,b,c,f,h,j
V_11_(%)	16.2 ± 4.4	27.4 ± 16.9	20.9 ± 5.0	27.5 ± 5.3	18.47 ± 4.53	a,b,c,f,h,j
V_12_(%)	7.4 ± 2.3	11.8 ± 9.0	10.5 ± 3.5	15.7 ± 3.9	11.17 ± 3.27	b,c,e,f,g,j
D_mean_(Gy)	7.8 ± 0.7	8.7 ± 1.1	7.3 ± 0.4	7.8 ± 0.8	5.5 ± 0.9	b,d,f,g,h,i,j
RA						
D_mean_(Gy)	8.9 ± 0.6	9.4 ± 1.0	9.0 ± 0.9	10.3 ± 0.9	8.3 ± 1.1	c,f,h,j
RV						
D_mean_(Gy)	6.0 ± 1.1	6.7 ± 1.8	5.3 ± 0.9	5.0 ± 1.0	2.8 ± 1.3	c,e,g,h,i,j
LA						
D_mean_(Gy)	7.8 ± 0.7	8.9 ± 1.3	7.5 ± 0.8	6.5 ± 1.0	4.2 ± 1.0	a,c,e,f,g,h,i,j
LV						
D_mean_(Gy)	7.9 ± 0.7	9.5 ± 1.6	7.6 ± 0.5	8.5 ± 0.8	6.2 ± 1.3	a,c,d,f,g,h,i,j

RA, right atrium; RV, right ventricle; LA, left atrium; LV, left ventricle; a, IMRT vs VMAT; b, IMRT vs HT; c, IMRT vs PT; d, VMAT vs HT; e, VMAT vs PT; f, HT vs PT; g, IMRT vs IMPT-2; h, VMAT vs IMPT-2; I, HT vs IMPT-2; j, IMPT-1 vs IMPT-2.

## Discussion

Unlike adult tumors, stage IV pediatric tumors usually have a satisfactory prognosis. For Wilms tumor patients, the survival analysis showed that the 16-year relapse-free survival (RFS) rate was 70%, and more than 80% of patients were expected to achieve 16-year overall survival (OS) (3). WLI is commonly employed in the treatment of pediatric malignancies, such as Wilms tumor, rhabdomyosarcoma and Ewing sarcoma, as part of the curative intent of the management of stage IV disease. Pediatric patients with sarcomas and pulmonary metastasis are usually treated with chemotherapeutic anthracyclines. In recent years, accumulating evidence has demonstrated that most of the above methods are correlated with adverse effects, including CHF and secondary malignant neoplasms (13). A few investigations have indicated that WLI is an important factor contributing to the development of heart failure in childhood cancer survivors (14–16). Furthermore, recent studies have indicated that irradiation of the heart can cause various disorders associated with the endocardium, myocardium, pericardium, coronary arteries, conduction system, and cardiac valves. Preliminary research has reported that a cardiac dose higher than 15 Gy is associated with cardiomyopathy or valvular disease (17). The Institute Gustave Roussy report indicated that the 20-year incidence of CHF was 18% after a heart dose >3.7 Gy and 9% after lower doses (18). Tukenova et al. studied 4,122 5-year survivors of a childhood cancer diagnosed before 1986 in France and the United Kingdom and confirmed that receiving radiation to the heart increased cardiovascular morbidity/mortality, with an estimated relative risk of 1.6 at a mean dose of 1 Gy (19). The American Wilms Tumor Study and Childhood Cancer Survivor Study showed that cardiovascular disease and secondary malignancies were the main causes of morbidity and mortality in long-term survivors (20, 21). Recently, a retrospective study of the pathophysiological observations of cardiovascular disorders in childhood cancer survivors linked anthracyclines (≥100 mg/m^2^) alone or combined with ≥15 Gy chest radiotherapy (RT) with poor OS, and children younger than 5 years old at diagnosis were vulnerable to radiotherapy-related adverse effects and an increased risk for cardiac complications (22). Therefore, reducing the adverse effects of radiotherapy is of great significance to the management of these populations (9).

The above findings emphasize the need to focus on normal tissue sparing when designing radiotherapy plans. Based on these therapeutic risk factors associated with cardiac disease, researchers have attempted to explore the values of strategies to reduce cardiac exposure using new radiotherapy techniques. Additionally, radiation-induced cancers are more common in children than in adults because of increased susceptibility to secondary cancers (23). Other very important organs around the lungs include the vertebral column, humerus, esophagus, liver, and spinal cord. Increased evidence has demonstrated that advanced radiotherapy techniques allow radiation oncologists to improve treatment, leading to maximal therapeutic efficacy with minimal adverse effects. A study published by Christina et al. confirmed the advantages of IP-AP/PA and VMAT techniques over standard AP/PA in normal tissue sparing (9). Kalapurakal et al. reported a significant decrease in the doses delivered to the OARs in the cardiac-sparing IMRT technique for WLI and confirmed the feasibility of this technique in a clinical trial consisting of 20 patients (24).

As an increasing number of pediatric patients have access to new forms of radiotherapy, efforts to improve heart exposure have followed. In this research, we assessed dose reductions to the heart and substructures with IMRT, VMAT, HT, and IMPT plans in the treatment of children undergoing WLI. Regarding the three photon plans, the results indicated that HT significantly lowered the dose to the heart and yielded the best coverage and homogeneity to the target structure. Additionally, the HT plan afforded superior dose sparing for the V_5_, V_6_, V_7_, V_8_, and D_mean_ of the heart and D_mean_ of the RV. Previous research showed that HT has the ability to conformally avoid reducing doses to normal tissues that are close to tumor-bearing regions, resulting in the superior capability of homogeneous dose distributions within targeted regions. HT has improved patient care through image-guided positioning and adaptive plans and prolonged the overall treatment times; thus, it represents both a novel radiation treatment device and an innovative means of delivering radiotherapy. More importantly, unlike VMAT, HT has great flexibility in treating multiple targets within a large volume in a simple setup. Moreover, IMRT demonstrated excellent conformity and displayed the most notable dose reductions in the V_9_, V_10_, V_11_, and V_12_ of the heart and D_mean_ of the RA. The VMAT plan was the least effective at sparing the heart and other normal tissues.

In recent years, with the development of radiotherapy technology, protons have gradually been used in the treatment of tumors. The major advantage of protons over traditional photons is that there is an obvious local high-dose region at the end of the dose range; this is referred to as the Bragg peak. The use of this property can ensure both a precise dose in the target area and low irradiation on the surrounding tissues and organs, improving the quality of life for cancer survivors, particularly children. As a result, a low-to-intermediate radiation dose may increase the risk of functional impairment as well as radiation-induced malignancies (25). From a purely physics focused point of view, the dose distribution of protons is, in most cases, superior to that of photons, although the lateral dose fall-off is worse for protons at higher energies than for photons (refer to Engelsman, this issue). In contrast, a proton beam does not experience the lateral penumbra widening that a photon beam experiences in the lung, a great advantage for PT.

When we first designed the IMPT-1 plan, we used both lungs as the PTV, similar to when we designed the photon radiotherapy plan, and found that PT is equivalent to photon radiotherapy in reducing the cardiac dose but not providing a dose advantage in cardiac protection. During PT, the peak part is aimed at the focus of the tumor, and the tumor receives the largest amount of radiation, while the normal cells in front of the tumor receive only 1/3 to 1/2 of the peak energy, and the normal cells at the back of the tumor essentially do not experience any radiation damage. Considering the anatomical positions of the heart and lung, regardless of the radiation field, the heart is surrounded by the target area, not the back of the whole lung (26, 27). Published studies have indicated that the effectiveness and degree of IMPT dose sparing to various OARs depend on the intracranial tumor location (28). Considering the anatomical relation between the whole lung and heart, we proposed a novel proton radiotherapy solution for children with WLI that has rarely been reported in previous studies. This solution significantly reduces the dose to the heart and explores the advantages of proton radiotherapy. Therefore, this approach may decrease the incidence of long-term complications associated with WLI. The future of pediatric radiation oncology research will determine patients who will benefit the most from PT.

Currently, IMPT is not widely applied in clinical practice, and it is sensitive to organ movement. Thus, this problem needs to be solved by combining respiratory gating techniques; however, these techniques are still in the research and improvement stage (27). Furthermore, current estimates of the benefit of PT over photon therapy based on toxicity reduction will be realized only when survivorship has been achieved. IMPT is limited by its technology and infrastructure, making it challenging to use in clinical applications; moreover, there are few proton centers in other countries, and the treatment is very costly. Currently, there are many dosimetry studies and small cohort or short-term follow-up studies. As a new technique, PT is an immature treatment plan for tumors with different shapes and locations. Different planning systems and different linear accelerator (LINAC) machines produced by other manufacturers should be studied in future investigations to overcome the variance between treatment facilities. With the improvement in the proton TPS and its physical properties, we believe that PT will benefit more patients.

## Conclusions

Our findings show that proton therapy, as a new radiotherapy modality that sums two PT plans and uses the unilateral lung as the PTV, is superior to the plan that uses the bilateral lung as the PTV for cardiac sparing in WLI. Considering the complex anatomical relation between target volumes and OARs, PT can provide a dose advantage for organs located outside the target area rather than within or surrounding the area. It is hoped that advances in PT plan design will lead to further improvements in radiotherapy approaches and provide the best treatment choice for individual patients.

## Data Availability Statement

The raw data supporting the conclusions of this article will be made available by the authors, without undue reservation.

## Ethics Statement

The retrospective research of the medical records was approved by Institutional Review Board of the Shandong Cancer Hospital for this analysis.

## Author Contributions

XS, JD, and YY designed the study and wrote the initial draft of the manuscript. XL, TS, and HW contributed to the design of the study and the analysis and interpretation of data and assisted in the preparation of the manuscript. JZ, HW, and XM contributed to data collection and interpretation, and critically reviewed the manuscript. All authors contributed to the article and approved the submitted version.

## Funding

This study was supported by the Key Support Program of Natural Science Foundation of Shandong Province (Grant No. ZR2019LZL017) and the Taishan Scholars Project of Shandong Province (Grant No. ts201712098 and No.tsqn201909140), and the Academic promotion program of Shandong First Medical University (Grant No. 2020RC003) and the National Natural Science Foundation of China (Grant No. 81901743).

## Conflict of Interest

The authors declare that the research was conducted in the absence of any commercial or financial relationships that could be construed as a potential conflict of interest.
